# Resolving the titer of murine cytomegalovirus by plaque assay using the M2-10B4 cell line and a low viscosity overlay

**DOI:** 10.1186/1743-422X-11-71

**Published:** 2014-04-18

**Authors:** Katherine A Zurbach, Toktam Moghbeli, Christopher M Snyder

**Affiliations:** 1 Department of Microbiology and Immunology, Jefferson Medical College, Kimmel Cancer Center, Thomas Jefferson University, 233 S. 10th St BLSB, rm 526, Philadelphia, PA 19107, USA

**Keywords:** Murine cytomegalovirus (MCMV), Plaque assay, M2-10B4 cells

## Abstract

**Background:**

Murine cytomegalovirus (MCMV) is increasingly used as an infectious model to investigate host-pathogen interactions in mice. Detailed methods have been published for using primary murine embryonic fibroblasts (MEFs) for preparing stocks and determining viral titers of MCMV. For determining the titer of MCMV by plaque assay, these methods rely on a high viscosity media that restricts viral spreading through the supernatant of the culture, but is also usually too viscous to pipet. Moreover, MEFs must be repeatedly generated and can vary widely from batch-to-batch in purity, proliferation rates, and the development of senescence. In contrast, the M2-10B4 bone marrow stromal cell line (ATCC # CRL-1972), which is also permissive for MCMV, has been reported to produce high-titer stocks of MCMV and has the considerable advantages of growing rapidly and consistently. However, detailed methods using these cells have not been published.

**Methods:**

We modified existing protocols to use M2-10B4 cells for measuring MCMV titers by plaque assay.

**Results:**

We found that MCMV plaques could be easily resolved on monolayers of M2-10B4 cells. Moreover, plaques formed normally even when cultures of M2-10B4 cells were less than 50% confluent on the day of infection, as long as we also used a reduced viscosity overlay.

**Conclusions:**

Overall, our protocol enabled us to use a consistent cell line to assess viral titers, rather than repeatedly producing primary MEFs. It also allowed us to start the assay with 4-fold fewer cells than would be required to generate a confluent monolayer, reducing the lead-time prior to the start of the assay. Finally, the reduced viscosity CMC could be handled by pipet and did not need to be pre-mixed with media, thus increasing its shelf-life and ease-of-use. We describe our results here, along with detailed protocols for the use of the M2-10B4 cell lines to determine the titer and grow stocks of MCMV.

## Background

Murine cytomegalovirus (MCMV) is increasingly used as an infectious model to investigate cytomegalovirus (CMV) biology, CMV-driven immune responses and host-pathogen interactions. Murine embryonic fibroblasts (MEFs) are the “gold standard” cell type for the growth of MCMV [1]. However, these cells are not immortalized and thus, must be produced repeatedly from freshly harvested mouse embryos. Generating batches of MEFs takes several weeks, including the 17 to 18 days of pregnancy it takes for the embryos to develop to the appropriate stage [1]. Moreover, in our hands, significant batch-to-batch variation has been evident. While many stocks of MEFs have grown robustly in culture and effectively produced infectious stocks of MCMV, other batches have grown slowly, failed to expand to useful numbers *in vitro* or produced low titers of MCMV. Thus, our experiments were often delayed by such variability. If new mice were needed to generate more MEFs, the delay could last several weeks or more.

The M2-10B4 cell line (ATCC # CRL-1972) is a bone marrow stromal cell line derived from a (C57BL/6J X C3H/HeJ) F1 mouse [2,3]. Previous work has shown that M2-10B4 cells support robust growth of MCMV, comparable to that of MEFs [4], and several reports cite the use of M2-10B4 to grow MCMV stocks (e.g. [5-7]). However, plaque assays to determine MCMV titer with M2-10B4 cells have not been described and the protocol for using M2-10B4 cells to grow viral stocks has not been described in detail.

In published protocols that detail MCMV plaque assays, a high viscosity media containing gum tragacanth or methyl-cellulose is used to restrict MCMV from spreading through the supernatant of the culture [1,8,9]. However, these overlays are too viscous to pipet and are usually pre-mixed with media, limiting the shelf-life of the overlay [1]. Other manuscripts note the use of alternative overlays such as Seaplaque (e.g. [10,11]), agar (e.g. [12]), agarose (e.g. [13]), or carboxy-methyl-cellulose (e.g. [14,15]), however detailed methods are not available. Of note, many viscosities of carboxy-methyl-cellulose (CMC) are available and the viscosity can vary from lot-to-lot. Reduced viscosity overlays have been described for other viral plaque assays [16], but not for MCMV.

Because of their robust growth and their reported ability to grow high-titer stocks of MCMV, we wished to use M2-10B4 cells to grow and measure the infectious titer of MCMV stocks. However, the lack of standardized protocols forced us to test cell numbers, time points and the viscosity of the overlay in a systematic way. In the end, we developed a robust protocol for using M2-10B4 cells that requires fewer cells to start the assay and a lower viscosity overlay than the previously published protocols. Although we are sure that other laboratories use variations of the protocols described below, the lack of detailed protocols necessitated a systematic exploration of the technical considerations. Our goal in producing this manuscript is to highlight the potential uses of M2-10B4 cells and to detail the protocol we settled upon to use these cells effectively. With the increased use of MCMV as a model of viral infection, we felt it was important to describe our approach and the results of our investigations.

## Results and discussion

### General plaque assay using subconfluent cells and a low viscosity overlay

**Figure 1:**
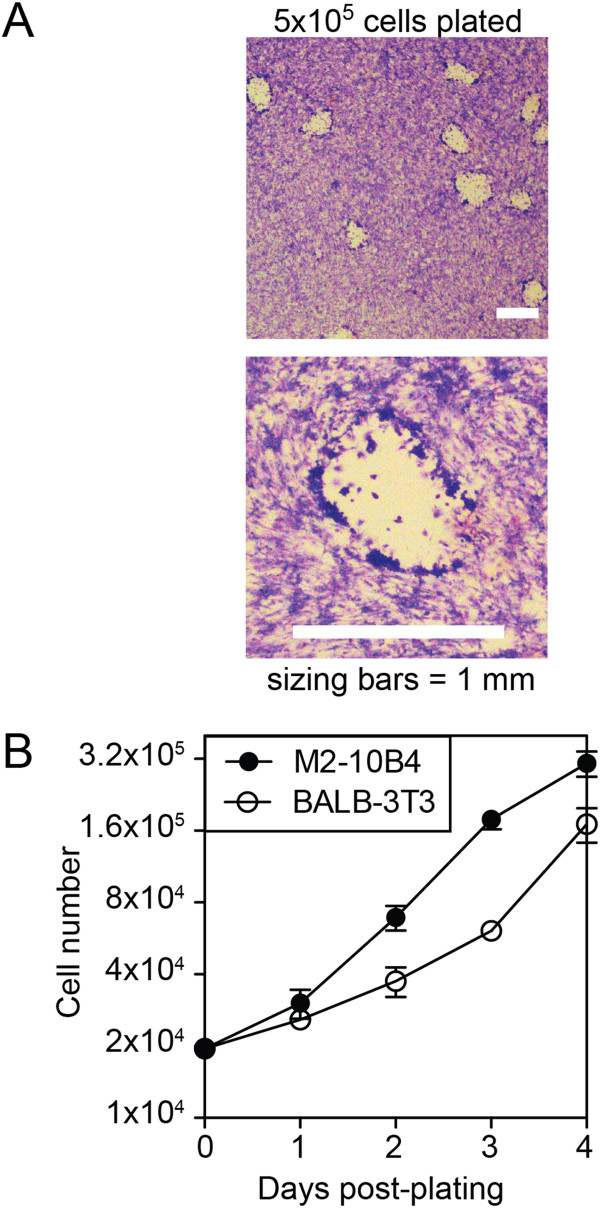
**M2-10B4 cells can be used to generate MCMV plaques. A)** Six-well plates were seeded with 5 × 10^5^ M2-10B4s one day before infection. Cells were infected with BAC-derived MCMV (MW97.01 [17]) for 1.5 hours, overlaid with high viscosity CMC mixture (1600 mPas final viscosity), and incubated for 6 days. Monolayers were fixed and stained with crystal violet solution as described. Representative images are shown. Sizing bars = 1mm in each image. **B)** M2-10B4 cells or Balb-3T3s were seeded in replicate wells and live cells were harvested and counted on the indicated days.

Previously described plaque assays for MCMV call for infecting a monolayer of fibroblasts. We use 6-well plates for plaque assays (surface area per well equal to 9.6 cm^2^) because it enables enumeration of a larger number of plaques per viral dilution. To determine the appropriate number of M2-10B4 cells to plate in each well of a 6-well plate we titrated their numbers. The following day, wells seeded with ≥ 5 × 10^5^ cells had become a confluent monolayer (not shown). After this overnight incubation, the monolayer was infected for the plaque assay. A detailed methods section is included below for a more thorough description. In brief, MCMV was serially diluted in media in 10-fold increments and 100 μl of diluted virus added to 1 mL of media in each well. The cells and virus were incubated together for 1.5 hours at 37°C, without centrifugal enhancement, before adding an overlay. We used an overlay of carboxy-methyl cellulose (CMC), which can have a range of viscosities from lot-to-lot. Our lot of CMC is 2110 mPas when diluted to 2% in H_2_O. We generated a stock solution of CMC with a viscosity of 8000 mPas (7.6%) and diluted this 1:4 in complete media (2000 mPas). Then, 4 mL of the CMC overlay was added to the 1 mL containing virus already in each well, for an expected final viscosity of 1600 mPas. The overlaid, infected cells were incubated for 6 days, the monolayer was fixed and stained with crystal violet, and plaques were visualized on a light box and/or via a dissection microscope. As shown in Figure 1A, when the monolayers were confluent at the start of the assay, viral plaques formed and were easily detected using this procedure.

There are several limitations to the procedure described above, however. First, the monolayer of M2-10B4 cells was apt to come off of the plate during the culture. These issues could be avoided by good husbandry of the cell line and instances of monolayer instability were typically traced back to cultures that were allowed to become > 50% confluent at some point during passaging. Second, pipetting the stock CMC (8000 mPas), was very difficult. Thus, the CMC had to be mixed with culture media in advance, limiting it's shelf-life. Finally, the assay required us to seed at least 3 × 10^6^ M2-10B4 cells per 6-well plate to generate monolayers. Even with the relatively rapid growth rate of M2-10B4 cells (Figure 1B), this required considerable lead-time to prepare each assay - either in growing cells or in maintaining large numbers of cultures - particularly when many plaque assays were needed simultaneously.

**Figure 2:**
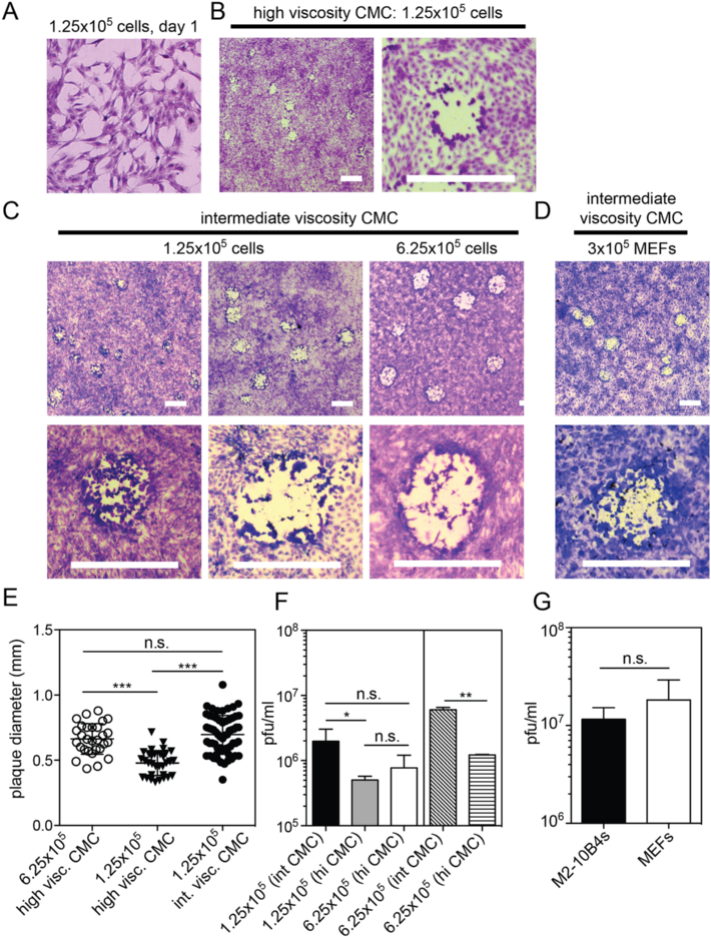
**Plaques can be resolved on sub-confluent monolayers of M2-10B4 cells with reduced viscosity CMC. A)** 1.25 × 10^5^ M2-10B4 cells were plated in 6 well plates and stained with crystal violet one day later. The representative image covers 1 mm^2^. **B)** 1.25 × 10^5^ M2-10B4s were plated in 6-well plates. One day later, cultures were infected with BAC-derived MCMV and overlaid with a high viscosity CMC as in Figure 1. Representative images show plaques 6 days after infection. Sizing bars = 1 mm. **C)** Either 1.25 × 10^5^ or 6.25 × 10^5^ M2-10B4s were plated and infected with MCMV as above except that cultures were overlaid with an intermediate viscosity CMC. Plaques were resolved 6 days later. Sizing bars = 1 mm. **D)** 3 x 10^5^ MEFs, generated as described [1], were plated, infected and overlaid as in “**C**”. Images show plaques 5 days later. Sizing bars = 1 mm. **E)** Plaque width was measured digitally, with a line drawn across plaque images that was converted to millimeters using the sizing bar. Each point represents a single plaque. (***p < 0.0001, as assessed by a student’s t-test). **F)** Cultures of M2-10B4s were infected with separate aliquots of a single viral preparation (left three columns, n = 2 to 5 assays per column) or a second viral preparation (right two columns, n = 2 assays per column). (*p = 0.015, **p = 0.005 as assessed by a student’s t-test). **G)** A single preparation of MCMV V70 [20,21] (kindly provided by Dr. Ed Mocarski) was assessed by infection of sub-confluent M2-10B4s (1.25 × 10^5^ cells plated, n = 3) or confluent MEFs (3 × 10^5^ cells plated, n = 3). Cultures were overlaid with intermediate viscosity CMC for comparison. Significance was determined by a student's t-test.

The considerations described above led us to test whether fewer cells and a lower viscosity overlay could be used. To this end, we began with subconfluent monolayers of M2-10B4 cells. Our previous cellular titration experiments revealed that 1.25×10^5^ M2-10B4 cells per well resulted in monolayers that were ~50% confluent 1 day after seeding (Figure 2A). When these monolayers were infected and overlaid with high-viscosity CMC, the resulting plaques were small and difficult to detect (Figure 2B). Moreover, the monolayer was noticeably less dense after 5 or 6 days of culture when compared to cultures that were confluent to begin with (not shown), resulting in reduced contrast between the plaque and the monolayer. Importantly however, under an intermediate viscosity CMC overlay (viscosity = 2400 mPas for the stock solution, 480 mPas final), the monolayer of cells grew to confluence, plaques were clearly visible, the average plaque size was restored and plaques were comparable to those obtained after infection of confluent monolayers of primary MEFs (Figure 2C, D and E). Indeed, plaques were easily counted within 5 days of infecting the cultures (not shown). The stock CMC at this intermediate viscosity (2400 mPas), although quite viscous, could be handled by pipet and was mixed with media shortly before it was added to the cultures. This enabled us to produce and sterilize bottles of CMC that were not pre-mixed with media, prolonging their shelf-life. Interestingly, we consistently detected slightly more plaques (although it was not always statistically significant) when intermediate viscosity CMC was used in the overlay, regardless of the starting cell number (Figure 2F). Most importantly however, under equivalent conditions, comparable viral titers were achieved with infections of subconfluent M2-10B4s and confluent MEFs (Figure 2G, representative of 3 assays covering 3 individually produced viral stocks). Likewise, comparisons of plaque assays performed using M2-10B4 and Balb-3T3 cells, our previous standard [18,19], resulted in comparable titers (not shown).

We have successfully used this protocol with non-recombinant laboratory strains of MCMV, namely Smith, V70 and K181 (Figure 2G and not shown), as well as to detect MCMV within salivary gland homogenates from infected Balb/c mice (not shown). Thus, our protocol is applicable to the measurement of recombinant or laboratory strains of MCMV derived from either tissue culture or salivary gland stocks. It is worth noting however, that organ homogenates were more often associated with loss of the monolayer during the culture period in our hands. To mitigate this, particularly when assessing MCMV in salivary gland homogenates, the homogenate was removed after 1.5 hours and the wells were gently rinsed at least 3 times with PBS before the addition of the CMC-overlay. In addition, we have been able to consistently generate plaques using M2-10B4s in 24-well plates (not shown). The surface area of a single well in a 24-well plate is approximately 1/5^th^ that of a well in a 6-well plate. Thus, reducing the starting cell number to 2.5×10^4^ or 1×10^5^ cells per well resulted in comparable subconfluent or confluent monolayers respectively. However, in our hands, 24-well plates have been 1.5- to 2-fold less sensitive than 6-well plates in determining viral titer as assessed by direct, parallel measurements of the same viral stocks (not shown).

**Figure 3:**
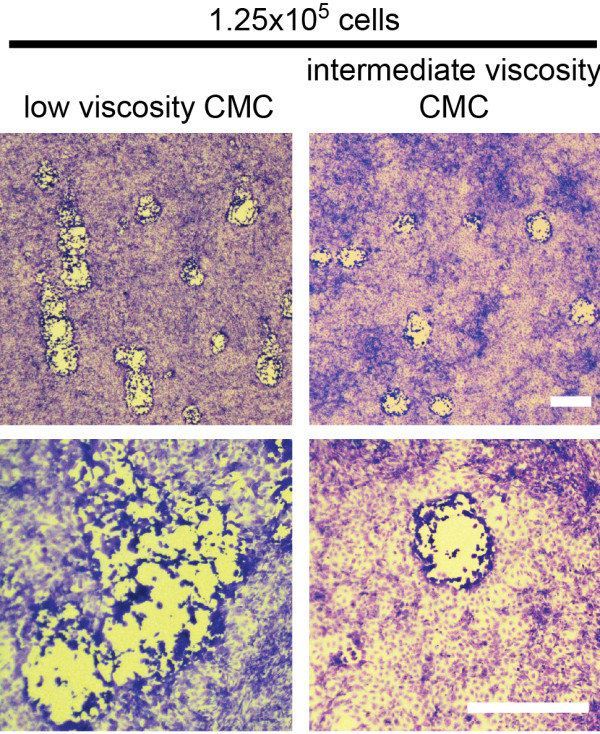
**Low viscosity CMC is not sufficient to prevent viral spread on M2-10B4 monolayers.** Monolayers begun with 1.25 × 10^5^ M2-10B4 cells (subconfluent) were infected and overlaid with low viscosity CMC (158 mPas final, left images) or intermediate viscosity CMC (480 mPas final, right images). Monolayers were fixed and stained 5 days after infection with BAC-derived MCMV. Sizing bars = 1 mm.

While CMC with a reduced viscosity was sufficient to prevent viral spread within the monolayer in all cases described above, it should be noted, that we could not reduce the viscosity of CMC indefinitely. Low viscosity CMC overlay (viscosity = 790 mPas for the stock solution, 158 mPas final) was unable to prevent the spread of MCMV and we observed comet-shaped plaques, which became particularly evident after day 4 (Figure 3).

Together, these data show: i) that viral plaques were readily detected on monolayers of M2-10B4 cells, ii) that infection of sub-confluent M2-10B4s, overlaid with reduced viscosity CMC, resulted in normal plaque development and faithfully reported on the infectious titer of MCMV stocks and iii) that an overlay with reduced viscosity was sufficient to prevent viral spreading within the culture.

### Additional Considerations - Care of M2-10B4 cells

Long-term culture and passaging may alter cellular phenotype and function. Throughout the described work, we used M2-10B4 cells passaged fewer than 30 times and cells were always passaged when the monolayer was ≤ 50% confluent. As noted above, proper care of the M2-10B4 cells was critical and we observed changes in the growth of M2-10B4 cells if they were allowed to become confluent prior to passage. Specifically, M2-10B4 cells tended to grow in clusters and lines, and cells were less spread out on the plate (not shown). This correlated with poor plaque development in subsequent assays and/or poor adherence of M2-10B4 monolayers to the culture dish for the duration of the 5 or 6 day assay (not shown). However, when M2-10B4 cells were carefully monitored during passage and not allowed to exceed 50% confluence at any time, plaques developed normally even when cells had been passaged > 50 times prior to the assay (not shown).

### Preparation of viral stocks

M2-10B4 cells have also been used to prepare stocks of infectious MCMV [5-7], although protocols have not been described in detail. To grow stocks of MCMV using M2-10B4 cells, as described in detail below, we typically seed 4 × 10^6^ M2-10B4 cells per plate in 15 cm dishes the day before the infection. Cells from a representative plate are counted one day later (to determine the approximate cell count after the overnight incubation) and all remaining plates are infected at a multiplicity of infection of 0.01. With this approach, all of the cells showed signs of viral infection (i.e. cytopathic effect, not shown) after 5 days. In our hands, infectious virus could be detected by plaque assay in both the supernatant from infected M2-10B4 cells, as well as the cell pellet from scraped monolayers (not shown). Thus, our protocol calls for combining the supernatant and homogenized cell pellet for concentration of the virus. Viral concentration is performed by ultra-centrifugation after removal of most cell debris by a low-speed spin. While this approach will not yield pure infectious viral particles, as are obtained by centrifugation through a 15% sucrose cushion [1], we routinely obtain infectious titers in the range of 10^7^ pfu/mL of BAC-derived, Smith and K181 MCMV.

## Conclusions

Primary MEFs must be produced repeatedly and can often vary markedly from batch-to-batch. In our lab, some batches of MEFs have ceased to grow shortly after isolation, necessitating long delays while we generated new batches of primary cells. MEFs can be immortalized using the 3T3 approach (e.g. serial passaging) and we have previously used such immortalized Balb-3T3s for many of our plaque assays (e.g. [18,19]). However, Balb-3T3s grow more slowly than M2-10B4s (Figure 1B) and thus required substantial lag times while we grew enough cells to generate monolayers.

With the approaches described here, we have obtained consistent results using M2-10B4 cells to grow stocks and determine the infectious titer of MCMV. Our data show that both confluent and sub-confluent monolayers of M2-10B4 cells can readily form MCMV plaques. Importantly, a reduced viscosity overlay medium was required to permit optimal plaque development when the assay was started with a sub-confluent monolayer (Figure 2). However, beginning with sub-confluent cells required less than 1/4^th^ of the number of cells and enabled us to maintain relatively few plates of M2-10B4 cells prior to the start of the assay. Moreover, comparable numbers of MCMV plaques were detected using M2-10B4 cells begun as sub-confluent monolayers and primary MEFs begun as confluent monolayers (Figure 2G). In our view, the use of overlays with reduced viscosities is an advantage over those with high viscosities since the solution is readily pipetted and does not need to be pre-mixed with culture medium. Moreover, the use of a reduced viscosity overlay may slightly increase the sensitivity of the plaque assay (Figure 2F).

We are certain that other labs use variations of the protocol described here. However, there was a relative lack of detailed information in the published literature and quick survey of published methods reveals evident lab-to-lab diversity in plaque assay protocols, particularly with respect to the overlay chosen [1,8-15]. Details about the viscosity and preparation of these overlays are often limited, including in our own previous work [18,19]. Thus, our goal in producing this manuscript was to both highlight the potential uses of M2-10B4 cells, as well as to detail our current protocols for comparison with the assays used by other labs working with MCMV.

## Methods

### Materials

1. Complete Growth Media: RPMI-1640 Medium with L-glutamine (Mediatech/Cellgro, cat #: 10-040-CV), supplemented with 10% FBS and 100 Units/mL Penicillin, 100 μg/mL Streptomycin

2. 1X PBS: 137 mM NaCl, 2.7 mM KCl, 5.4 mM Na_2_HPO_4_-dibasic, and 1.8 mM KH_2_PO_4_-monobasic, pH 7.4

3. 0.25% Trypsin-EDTA (Life Technologies, cat # 25200)

4. Intermediate Viscosity Carboxymethylcellulose (2400 mPas for the stock solution): Carboxymethylcellulose has a wide range of viscosities. The work described here used a batch from Calbiochem (cat #: 217274) with a viscosity of 2110 mPas when suspended at 2% (w/v) in water. Thus, to achieve a CMC stock with a viscosity of 2400 mPas, we dissolved 6.82 g of CMC in 300 mL.

i. Mix 180 mL 1X PBS with 120 mL distilled/deionized water, and place on heated stir plate with stir bar.

ii. Calculate the necessary percent of High Viscosity Carboxymethylcellulose needed to achieve a stock viscosity of 2400 mPas (6.82 g in our case) and add slowly to the stirring PBS/water solution.

iii. Stir, covered and heated, until all CMC is dissolved. This may take several hours, especially if the CMC has clumped together. Heat can be turned off and CMC stirred overnight.

iv. Aliquot and autoclave. Allow to cool fully before use.

5. Carboxymethylcellulose overlay media: Dilute CMC, prepared as above, 1:4 with complete growth media.

**Note:** When preparing the CMC overlay, take care to fully dispense the viscous mixture from the pipet. Typically, we add the CMC to the media and use the diluted mixture to rinse the pipet. In addition, be sure to swirl the solution to ensure complete mixture of media with the viscous CMC.

6. 10X Crystal Violet**:** 1g Crystal Violet (EMD, cat #: 192-12), 10 mL 37% Formaldehyde, 90 mL 1X PBS,

i. Add 1g Crystal Violet to the PBS/Formaldehyde solution, and stir, covered, on a warm hot plate in a fume hood until crystal violet is dissolved, usually several hours.

ii. Vacuum filter through a 0.45 μm filter to remove any remaining crystals.

7. 1X Crystal Violet: Dilute 10X crystal violet from above 1:10 with PBS.

8. Additional standard materials (detailed in the methods below):

15 cm plates

multi-well dishes

cell scrapers

tight fitting dounce

ultracentrifuge rotor and tubes

### Growing M2-10B4 cells

M2-10B4 cells are grown in complete media at 37°C in 5% carbon dioxide. We typically maintain M2-10B4 cultures in tissue culture dishes with a 15 cm diameter and split the cells as described below, every Monday, Wednesday and Friday to maintain cultures that are less than 50% confluent. We have had success growing M2-10B4 cells on tissue culture plastic from several manufacturers (e.g. Corning Life Sciences cat #: 353002 or NEST biotechnology cat #: 715001).

1. Remove the culture media and rinse cells 1X with PBS before adding enough 0.25% Trypsin-EDTA to cover the bottom of the plate.

2. After the cells round up, quench the trypsin with 1 volume of complete media and vigorously pipet the cells from the plate. Spin the cells at 500 × g for 7 to 10 minutes.

3. Resuspend the cells in 1 mL of complete media and count.

4. If cells are to be harvested (or re-split) 2 days later, we typically re-plate 3 × 10^5^ cells in 15 cm tissue culture dishes. If cells are to be harvested 3 days later, we typically re-plate 2 × 10^5^ cells.

### Viral plaque assay

#### Day before infection (Day -1)

1. Determine the number of 6-well plates you plan to infect. You will need one plate for each virus being titered, plus an additional plate for a standard control. It is possible to titer 2 viruses in the same 6-well plate, using 3 wells per virus, if you know the approximate range of titer expected.

2. Rinse M2-10B4 cells and add 0.25% Trypsin-EDTA to each plate to dislodge the cells for counting.

3. Add 2 mL of Complete growth media to each well of a six well tissue culture plate, and rock to distribute the medium.

4. Add 1.25 × 10^5^ cells per well for a sub-confluent monolayer or 5 - 6.25 × 10^5^ cells per well for a confluent monolayer. Incubate at 37°C, 5% CO_2_ overnight.

#### Day of Infection (Day 0)

##### Prepare the cells

1. Look at all plates of M2-10B4s under the microscope to make sure the cells look evenly distributed and the plates are ~50% confluent (for 1.25 × 10^5^ cells per well) or confluent (for 5 - 6.25 × 10^5^ cells per well). We do not use plates in which the cells are unevenly distributed or have an unusual morphology.

2. Pipette off all media and add 1 mL of pipetted media back to each well. Be careful not to let the cells dry out during this step.

**Note:** 1 mL of media was chosen as a volume that just covers the monolayer in a 6-well plate, thus preventing the cells from drying out during the incubation with virus (see below). However, if your incubator is not level, you may get areas that are not well covered by the media during the next steps and periodic rocking or turning the plates may be necessary to prevent cell drying or uneven infections.

##### For quantifying viral stocks

1. In a 96-well plate add 180 μL of media to each well for which a viral dilution is needed. We begin viral titrations at a 10^-1^ dilution. For high titer viral preparations, we typically test dilutions from 10^-2^ to 10^-6^ or 10^-3^ to 10^-7^.

2. Thaw viruses to be titered as well as an aliquot of a control virus (we use a low titer stock of MCMV, distributed and frozen in 30 μl aliquots).

3. Add 20 μL of each virus to the first well of a column in the 96-well plate containing 180 μL of media. This is the 10^-1^ dilution. Mix thoroughly, remove 20 μl with a fresh pipet tip, and add to the next well for a 1:10 serial dilution. Continue diluting the viruses serially in this way.

**Note:** Use new tips at every step through the dilution process. Virus will carry over through these serial dilutions, altering the accuracy of your dilutions.

##### For quantifying virus in organ homogenates

since viral titers tend to be much lower in organ homogenates, we often use a 3-fold serial dilution series.

1. In a 96 well plate, add 120 μl of media to each dilution well for each organ being tested.

2. Thaw the organ homogenates and add 60μl to each well. This is a 1:3 dilution. Dilute serially 1:3 as above.

##### Infect the 6-well plates

1. Label 6-well plates with the virus and dilutions.

2. Add 100 μL from each desired dilution of virus to individual wells in the 6-well plate. Repeat for each virus to be titered. It is a good idea to leave at least one well on one of your plates uninfected, to assess monolayer integrity in the absence of plaques.

3. Rock plate gently to distribute virus and then incubate at 37°C for 1.5 hours.

**Note:** We have had problems getting nice monolayers of M2-10B4 cells on some brands of 6-well plates for unknown reasons. Currently we use Thermo Scientific Nunc plates (NunclonDELTA multi-dishes, cat # 140675).

##### CMC overlay

1. While plates are incubating, warm CMC and complete media to 37°C in a water bath. Warming the stock of CMC will increase the ease of pipetting.

2. Determine the total volume of overlay needed for your plates (4 mL per well) and dilute the appropriate amount of CMC stock 1:4 with complete media (1 mL CMC + 3 mL complete medium) to generate the overlay.

3. After 1.5 hours of incubation, add 4 mL of the CMC/media overlay to each well. Alternatively, the media containing virus can be removed from each well and gently replaced with 4 mL of CMC mixture.

**Note:** If measuring virus from organ homogenates (in particular the salivary gland or liver), it may be necessary to rinse the wells with sterile PBS to avoid toxicity to the M2-10B4 monolayer.

4. Incubate at 37°C for 5 to 6 days.

**Note:** We have found that monolayers of M2-10B4s that are confluent at the start of the culture survive better if the plates are not disturbed during the 5 to 6 day incubation.

#### Day 5 or 6 Post Infection

##### Fix and Stain the Monolayer to Reveal Plaques

1. Remove plates from the incubator and check that the monolayer is intact by brief examination with a microscope. The plaques can also be assessed at this time.

2. Remove supernatant.

3. Rinse all wells gently with ~2 mL PBS. Repeat once more to remove all remaining CMC from the wells.

**Note:** At this step, prior to fixation, the monolayer may be fragile. We rinse the wells by angling the plate and dispensing PBS from a serological pipet onto the side of the well, using the slowest setting on the pipet and careful control of the pressure.

4. Invert plates on absorbent pad to allow excess PBS to run down the sides of the wells.

**Note:** allowing your monolayer to remain immersed in PBS for prolonged times while additional plates are rinsed may result in the cells detaching from the plastic.

5. Add 1 mL Crystal Violet to each well and incubate until the desired stain intensity is achieved (usually 2-5 minutes, but the time can vary with the batch of 10X Crystal Violet and how much was undissolved at the time of filtration).

**Note:** If undissolved crystal violet crystals are problematic, the 1X stock solution can be briefly warmed in a microwave, hot plate or waterbath. Do not add overheated crystal violet solution to your cells. The bottle should feel warm to the touch, but not so hot that you can’t hold it without burning your hands. Alternatively, the 1X solution can be re-filtered.

6. Rinse each well with ~3 mL distilled/deionized water (can be from a tap if the flow is slow enough), flick off and repeat once more.

7. Plaques can be counted immediately, or plates can be dried upside down on absorbent pads.

##### Calculating the Viral Titer in Plaque Forming Units (pfu) per mL

1. Obtain a raw plaque count: To get the most accurate titers, we typically count MCMV plaques in any well that has between 10 and 150 individual MCMV plaques. We verify plaques with a dissection microscope.

2. Convert the raw count to pfu/mL: The viral dilution series was not carried out in 1 mL of volume, and only a fraction of 1 mL was used to infect the monolayer (see step 2 of "Infect the 6-well plates" above). Thus, the raw plaque count (pfu) must be converted to pfu/mL by accounting for the fraction of 1 mL used. In the protocol described, we added 100 μL of diluted virus (or 1/10^th^ of 1 mL) to each well. So the raw plaque count obtained from a well infected with 100 μL of diluted virus must be multiplied by 10 to convert it to pfu/mL. If you use a smaller or larger volume of diluted virus to infect the monolayer, this volume should be accounted for in the calculation of pfu/mL.

3. Determine the titer of the viral stock: Multiply the pfu/mL by the dilution factor used to infect the individual well used for counting. For example: the pfu/mL calculated from a dilution of 10^-5^ must be multiplied by 10^5^ to determine the pfu/mL of the stock.

4. Final titers for viral stocks are an average of 3 separate plaque plates from 2 - 3 separate assays (i.e. replicates performed on separate days).

### Preparing Crude Stocks of MCMV

1. (Day -1) Trypsinize all cells as described above, count, and replate 4 × 10^6^ cells into new 15 cm plates.

2. (Day 0) Pick one representative plate and count it as above, then infect the remaining plates with an multiplicity of infection (moi) of 0.01 (i.e. 1 pfu for every 100 cells).

#### Once cells have all developed visible cytopathic effect (usually 4-5 days post infection)

1. Scrape all plates with a cell scraper and collect supernatant and cell debris in 50 mL conical tubes on ice.

2. Rinse all scraped plates with 10 mL PBS, using the same 10 mL to rinse each plate, and add to collected lysate.

3. Spin all tubes in a table-top centrifuge at 2600 × g for 10 minutes at 4°C to pellet the cells and cell debris.

4. Pool all supernatant and keep on ice.

5. Resuspend all cell pellets/debris in 10 mL media (use same 10 mL for all tubes).

6. Dounce debris on ice 15 to 20 times, using a sterile tight-fit dounce. Add dounced debris to a conical tube and spin in table-top centrifuge at 2600 × g for 10 minutes at 4°C. This will release some (but not all) virus still stuck to cells.

7. After centrifugation, remove the supernatant from the dounced fraction and add to the supernatant collected from the first spin, currently on ice.

**Note:** We typically resuspend the remaining cell pellet in 500 μl of media for each plate of the starting culture and aliquot it into several tubes. This can be used for later assessment by plaque assay to determine how much infectious virus remained with the cell pellet.

8. Distribute the collected supernatant into ultracentrifuge tubes and balance. We use an SW 32Ti swinging bucket rotor for this step (cat #: 369650) and Beckman thin wall ultracentifuge tubes (cat #: 344058). Spin in the supernatant at 50,000 × g for 1 hour at 4°C.

9. At the end of the centrifugation step, you should see a pellet at the bottom of each tube. Carefully remove supernatant from all tubes and resuspend pellets in 500 μl of complete media per initial plate of the culture. For example, virus from 5 infected 15 cm plates will be resuspended in 2.5 mL.

**Note:** We typically save some of the supernatant from this spin and aliquot it into several tubes. This can be used for later assessment by plaque assay to ensure that most virus was collected from the supernatant during the ultracentrifuge step.

10. Dounce the resuspended virus 10 times on ice to assure proper resuspension. You can use the same dounce that was used in Step 6.

11. Aliquot 100 - 200 μl of virus into screw cap cryovials and freeze at -80. Be sure to include at least 3 aliquots of 30 μl for plaque assays to assess infectious titer of the stock.

## Abbreviations

CMV: Cytomegalovirus; MCMV: Murine cytomegalovirus; Pfu: Plaque forming unit; MEF: Murine embryonic fibroblast.

## Competing interests

The authors declare that they have no competing interests.

## Authors’ contributions

KAZ and CMS designed the experiments. KAZ and TM performed the experiments. CMS wrote the manuscript. All authors read and approved the final manuscript.
